# Provider-Initiated HIV Testing for Migrants in Spain: A Qualitative Study with Health Care Workers and Foreign-Born Sexual Minorities

**DOI:** 10.1371/journal.pone.0150223

**Published:** 2016-02-25

**Authors:** Barbara Navaza, Bruno Abarca, Federico Bisoffi, Robert Pool, Maria Roura

**Affiliations:** 1 ISGlobal, Barcelona Centre for International Health Research (CRESIB), Hospital Clínic - Universitat de Barcelona, Barcelona, Spain; 2 Centre for Social Science and Global Health, University of Amsterdam, Amsterdam, The Netherlands; University of Athens, Medical School, GREECE

## Abstract

**Introduction:**

Provider-initiated HIV testing (PITC) is increasingly adopted in Europe. The success of the approach at identifying new HIV cases relies on its effectiveness at testing individuals most at risk. However, its suitability to reach populations facing overlapping vulnerabilities is under researched. This qualitative study examined HIV testing experiences and perceptions amongst Latin-American migrant men who have sex with men and transgender females in Spain, as well as health professionals’ experiences offering HIV tests to migrants in Barcelona and Madrid.

**Methods:**

We conducted 32 in-depth interviews and 8 discussion groups with 38 Latin-American migrants and 21 health professionals. We imported verbatim transcripts and detailed field work notes into the qualitative software package Nvivo-10 and applied to all data a coding framework to examine systematically different HIV testing dimensions and modalities. The dimensions analysed were based on the World Health Organization “5 Cs” principles: Consent, Counselling, Connection to treatment, Correctness of results and Confidentiality.

**Results:**

Health professionals reported that PITC was conceptually acceptable for them, although their perceived inability to adequately communicate HIV+ results and resulting bottle necks in the flow of care were recurrent concerns. Endorsement and adherence to the principles underpinning the rights-based response to HIV varied widely across health settings. The offer of an HIV test during routine consultations was generally appreciated by users as a way of avoiding the embarrassment of asking for it. Several participants deemed compulsory testing as acceptable on public health grounds. In spite of—and sometimes because of—partial endorsement of rights-based approaches, PITC was acceptable in a population with high levels of internalised stigma.

**Conclusion:**

PITC is a promising approach to reach sexual minority migrants who hold high levels of internalised stigma but explicit extra efforts are needed to safeguard the rights of the most vulnerable.

## Introduction

Despite progress towards universal HIV treatment access, the uptake of HIV testing has remained low in areas with generalised epidemics [1] and high-income countries with epidemics concentrated in men who have sex with men (MSM), injecting drug users (IDU), sex workers (SW), and migrants [2]. Recent estimates report that about a half of people with HIV remain unaware of their HIV status [3]. This is particularly disturbing in a context where the adoption of a *Test and Treat* approach—implying frequent universal testing and immediate initiation of antiretroviral treatment (ART)—has been heralded as the solution to HIV transmission [4]; and challenges the achievement of the ambitious 90-90-90 targets proposed by UNAIDS: by 2020, 90% of people with HIV should be aware of their infection, 90% of people diagnosed with HIV should be linked to antiretroviral treatment (ART), and 90% of those on ART should adhere and have undetectable levels of HIV in their blood [5].

The determination to increase HIV test coverage and to reduce missed opportunities for earlier diagnosis has lead to a major switch in HIV testing policies from the traditionally patient-initiated Voluntary Counselling and Testing approach (VCT) to Provider-Initiated Testing and Counselling (PITC) where health providers (HP) routinely offer an HIV test to all health service users (universal approach) or those meeting specific symptomatic, behavioural or country-of-origin criteria (targeted approach) [6–10].

While most countries with generalised epidemics have quickly adopted universal PITC policies [11–14], in high income countries with concentrated epidemics, universal testing recommendations are being issued more progressively. The United States pioneered the approach with the revised 2006 *Centers for Disease Control and Prevention* (CDC) HIV testing guidelines, calling for routine HIV screening amongst adults in all health care settings with HIV prevalence above 0.1% [15]. Two years later, UK guidelines recommended the systematic offer of an HIV test to patients aged 15–59 years registering in general practice in areas with HIV prevalence above 0.2% [16]. In 2009, the *French National Authority for Health* recommended untargeted routine screening of the 15–70 year-old population [17]. More recently, the *Spanish Ministry of Health* has also issued recommendations to expand the systematic offer of HIV testing at health facilities beyond symptomatic and behavioural criteria [18].

The global switch from VCT to PITC approaches has been accompanied by some ethical controversy [19–23]. Besides the risk of undesirable social consequences derived from an HIV+ diagnosis, it has been argued that the social status of medical professionals and tendency to obey authority could compromise the voluntariness that should underpin informed consent procedures [22,24–26]. Targeted PITC for migrant populations disproportionally affected by HIV has also been questioned: the selective offer of an HIV test ultimately based on skin colour and/or other physiognomic characteristics may be “epidemiologically tempting” [27,28] but there are worries that it is potentially discriminatory. For example, the *African HIV Policy Network* has raised concerns about the potentially stigmatising effects derived from screening populations for HIV on the basis of prevalence data [29] and the *UK National AIDS Trust* has highlighted the importance of scaling up HIV prevention for ethnic minorities, but in a way that prevents people from feeling “targeted” or “singled out” as a risk group for HIV infection [30]. In Belgium, targeted PITC for sub-Saharan migrants was seen as discriminatory practice and HPs feared accusations of racism. Many reported that testing undocumented patients who would subsequently not be able to access treatment was unethical [31]. In the UK, HPs thought that ethnic minority users would perceive their offer to test for HIV as a moral judgement if the universal nature of the approach was not clear [32].

Migrants often face overlapping vulnerabilities where ethnicity, socio-economic, and legal disadvantages operate synergistically to multiply the potential detrimental effects of a positive diagnosis [33]. This can be further exacerbated in contexts of economic downturn, with shrinking health care and social services budgets. In Spain, as part of the austerity measures that accompanied the economic recession, a decree-law in 2012 excluded undocumented migrants from full healthcare entitlements [34], which raises concerns about the humanitarian and epidemiological consequences of restricting care for the most vulnerable at times when it is most needed [35–37]. More recently, irregular migrants’ entitlements to health care have been partially reinstituted in this country [38].

A significant percentage of MSM in Europe with HIV+ diagnoses are foreign-born [39,40]. Individuals affected by both ethnic and sexual orientation discrimination have been reported to “posses a dual minority status” [41] and may be particularly vulnerable to HIV infection [42]. However, migrant MSM often remain “invisible” in discussions about HIV [27].

In Spain, Latin America is the most common region of origin amongst foreign-born HIV cases [39,43] and LA transgender women (TW)—often grouped as part of the broad MSM population but with distinctive characteristics [44,45]—bear an extremely high burden of the disease [46], with prevalence rates above 17% [47].

The success of PITC in identifying new HIV cases relies on its effectiveness in reaching individuals most at risk [48]. As PITC is increasingly promoted [16–18,49] it is important to assess its effects on populations facing overlapping vulnerabilities, including MSM and transgender females of foreign origin, undocumented migrants and persons who work in stigmatized occupations such as sex work [50]. Amidst concerns over the suitability and sustainability of PITC [31,51], careful assessments of users’ and providers’ views are needed to monitor adherence to the principles underpinning the rights-based response to HIV: Consent, Counselling, Connection to prevention, care and treatment, Correct test results, and Confidentiality. According to these principles, all HIV testing services should include pre-test information, post-test counselling, linkage to appropriate HIV prevention, care and treatment services, quality HIV testing, and accurate test results and diagnosis [49].

This qualitative study examines HIV testing experiences and perceptions about PITC amongst MSM and transgender females of LA origin as well as HPs’ current practice offering HIV tests to migrants in Spain. We provide empirical qualitative data to contribute to current debates on the adequacy of PITC to identify new HIV cases with specific attention to the World Health Organisation’s (WHO) essential 5Cs principles. Our aim is to inform HIV testing policies in Spain and other countries that are implementing PITC approaches where HIV disproportionally affects migrants.

## Methods

This qualitative study consisted of 32 in-depth interviews and 8 group discussions with a total of 38 MSM and TW originating from 14 different Latin American (LA) countries, as well as 21 health professionals providing care to migrant populations. Data collection took place between January 2013–March 2014 in Barcelona and Madrid, the two cities hosting the largest numbers of Latin American migrants in Europe.

A multi-disciplinary team of researchers composed of a sociologist (MR), two anthropologists (RP, FB), a cultural mediator (BN) and a clinician (BA) worked on the joint development of recruitment procedures, data collection tools, identification of emerging issues, refinement of study guidelines, and interpretation of results. The cultural mediator (BN) and the clinician (BA) underwent a 3-week course on qualitative methods taught by the PI (MR) with a focus on interviewing skills and participatory research attitudes and skills.

HPs were purposefully recruited from facilities serving high percentages of migrants and included professionals working in different roles (nurses, clinicians, managers) and types of services (community-based, primary care, specialised care). LA migrants who had ever tested for HIV were recruited with the help of community-based workers and selected to maximise diversity in terms of sex, age and country of origin (Table 1).

**Table 1:** Characteristics of study participants (N = 59).

**LATIN AMERICAN MIGRANTS**	**N = 38**
**Sex**	
Male	25
Female (transgender)	13
**Age**	
<30	18
30–49	13
>50	7
**Known HIV+ status**	8
**HEALTH PROVIDERS**	**N = 21**
**Sex**	
Female	14
Male	7
**Level of care**	
Primary	4
Specialised	14
Community-based	3
**Occupation**	
Clinician	11
Nurse	7
Community-based worker	3
**TOTAL PARTICIPANTS**	**59**

We developed five different field-work guidelines carefully tailored to gather insights from various types of participants (professionals working at health facilities, community-based workers, users) through different interview modalities. These included lightly structured individual interviews that employed time-lines to recall testing trajectories, semi-structured interviews, and participatory group discussions that employed a matrix covering various dimensions of diverse testing modalities. The initial guidelines where developed by BA (for facility-based health workers) and BN/FB (for users and community-based professionals). The final guidelines were refined through several rounds of comments and various meetings with the whole data collection team.

Recordings of interviews and group discussions were transcribed and reviewed when necessary. All the field workers filled-in debriefing forms after each of the activities, which they then discussed with the principal investigator (MR). The team met periodically throughout the whole data collection process in order to identify timely emerging topics and subsequently adapt data collection tools within an iterative research design, in which the data collected informed subsequent stages of the research process. As the first transcripts became available the PI provided individualised feedback to each field-worker, fostering self-reflexivity and highlighting the importance of posing follow-up questions to allow for greater depth of data and further investigation of emerging hypothesis with due attention to disconfirming evidence. All the transcripts and notes were imported into the qualitative software package Nvivo-10. We developed a coding framework to systematically examine the pros and cons of different HIV testing dimensions and modalities. The dimensions analysed were based on the 5Cs WHO principles and included consent, counselling, connection to treatment, correctness of results and confidentiality. The modalities were PITC, VCT, community-based testing (mobile units), pharmacy-based and self-testing. The coding framework was systematically applied to all the data and flexibly adapted to account for emerging themes. The senior author (MR) coded and analysed the data, and interpreted the findings with input from the rest of the team.

Ethical approval was obtained from the Hospital Clinic-University of Barcelona Ethical Committee Board (CEIC). At the recruitment stage all prospective participants were informed in detail about the overall aim of the project, the approach used and their expected role. Because we recruited participants regardless of their HIV status there was no risk of involuntarily disclosing individuals' serological profiles (although some participants spontaneously disclosed to researchers their HIV+ status). We administered informed consent procedures in a language that suited the participants' level of understanding and verbal consent was obtained from all participants. When consent to record was not provided we took detailed notes. Sharing of personal information during group sessions was discouraged and we stated explicitly the boundaries of the potential support to be offered by the project. No photos of individuals were taken without consent. We did not provide any remuneration. Minors under the age of 18 were not included in the study population. As this study carried a minimal risk, verbal consent and/or attendance and participation in the activities was considered sufficient proof of consent by the ethical committee board.

## Results

### Acceptability of PITC

The offer of an HIV test as part of routine medical encounters was generally perceived in positive terms. Many HPs reported that the sustained, repeated contact with users helped them to progressively build trust and sensitise them to the advantages of testing for HIV. Many valued PITC as an effective way to identify HIV+ cases at earlier stages of infection and especially among heterosexual persons who did not fall into any *risk group* category. However, in practice some HPs decided who was offered an HIV test based on their own perceptions of an individual’s risk, and this sometimes relied on observable indicators of socio-economic status and ethnicity.

*He was a man with an acceptable economic level*, *middle-class*, *forty years old*. *He had a job*, *was from here*, *had not been to prison*, *and did not take drugs*. *Because he had*, *let’s say*, *a good appearance*, *I did not even think of HIV*

[HP 8]

Most users appreciated the offer of an HIV test as a way of avoiding the embarrassment of asking for it and being qualified as a “gorrón” (abuser of the system) and/or implicitly recognised as gay/promiscuous.

If they don’t offer you the HIV test and you have to ask for it you feel like a beggar

[User 5, TW]

For many users, health facilities away from the MSM community were more appropriate HIV testing venues than mobile units where “anyone can see you coming in” as well as leisure spaces such as saunas, discos or gay pride events.

*I feel better entering the door of my primary care practitioner because everybody goes there*, *for a flu*, *for anything*. *Instead*, *when you enter a mobile unit it is clear that you go there because of HIV*. *I am not as worried about the infection as for the fact that if I get tested I will seem promiscuous*. *If I wasn’t*, *I wouldn’t need an HIV test*

[User 10, MSM]

Still, several expressed concerns about being discriminated against at health facilities because of their sexual orientation or HIV status and reported marked preference for community-based services where they were treated as “normal persons”.

*You can see those looks amongst nurses or doctors*. *They turn back to look at you and that is why many people don’t want to test at health facilities and prefer to go to a community-based association where they already know you and see you as a normal person*

[User 3, TW]

While the offer of HIV testing at health facilities was generally viewed in positive terms there were some concerns about its operational implementation and in particular, its adherence to the 5Cs principles underpinning the rights-based response to HIV.

### Consent: “It depends a lot on each provider”

The majority of HPs were fully aware of the importance of informing users appropriately and obtaining their consent prior to testing for HIV. However, attitudes and procedures varied dramatically across settings. These depended largely on HPs’ views independently of existing regulations and protocols. For most professionals, obtaining consent and not pressuring users was of paramount importance but several said that informed consent procedures were an unnecessary obstacle and a few admitted to have conducted HIV tests without asking for consent. One described it as common practice at his place of work and another explained that users could be “cheated a bit” when a test for which consent had not been sought turned out to be positive.

*I have seen many different ways of dealing with it*: *some say straightaway that amongst all the tests performed they will include HIV*. *Other HPs*, *when they see that the test result is HIV+ and they had not asked for consent*, *then they say to the patient that they are going to test him for HIV*. *It is after they already have the result that they tell the patient*. *In reality it is like cheating patients a bit*. *I am not saying this is generalised but there are many different ways of dealing with it*

[HP 15]

*It depends*. *Probably many doctors don’t ask for consent*. *My boss says we have to ask for it but no one does*. *Or you ask for it in a disguised way*. *Otherwise you get people worried*

[HP 18]

Compulsory testing was generally deemed unacceptable for users, but several reported that regular compulsory testing and HIV screenings at airports and workplaces were acceptable and even desirable measures. Two transsexual sex workers detailed that in their countries of origin the police would come with a list asking for their health cards and “punishing the girls” who had not visited the doctor, forcing them to test and undergo treatment for STIs including HIV. This was acceptable to them because “people there are very *pasota* (careless)*”* and such measures helped to keep them and the rest of population healthy. Other participants reported that they had undergone compulsory HIV testing as part of pre-migration or work recruitment procedures.

*It was a request of the company that hired us to come and work here*. *We had to undergo a general medical check*, *which included everything*, *including HIV*. *I don’t know if people with HIV+ results were allowed to get the job and enter the country*

[User 21, MSM]

*HIV tests should be compulsory even if it is a bit discriminatory*. *It is hard to say*, *but that’s the way it is*. *In the long term it will be good for him because the earlier he is attended*, *the better*

[User 17, MSM]

### Counselling: “I don’t want to have a questionnaire done to me”

Pre-counselling was often skipped or took the form of a behavioural survey that HPs perceived to be unreliable because users would “lie and lie” to prevent the embarrassment of disclosing intimate behaviours. In turn, being asked “an endless list of questions” discouraged some users from testing.

*They gave me 20 minutes to fill in something*. *Then they started to explain to me how you can get infected*. *They asked whether I consumed drugs*. *I said no*. *Whether I injected drugs*. *I said*, *no*. *Then they continued to ask whether I had done that*, *and I said yes*, *yes*, *yes*, *no*, *no*, *no*, *yes*, *yes*, *yes*, *no*, *no*

[User 4, MSM]

Skipping post-counselling sessions was also common and this was perceived positively by many users and providers who saw it as ineffective at promoting safer sexual behaviours. This was particularly the case amongst repeated testers who already knew “the sermon”.

*It is a sermon*. *They already know it*. *It is to reinforce knowledge and make them think about it again but they pay no heed to it*. *If a person comes back several times over the years with various infections*, *it means that no matter how many times you explain it [it still doesn’t have any effect]*. *I don’t know*. *I also get lost*. *I don’t know where I am going*. *Something is not working*, *that is for sure*

[HP 9]

We nonetheless identified important gaps in knowledge in a population originating from countries where “people still panic about HIV”. The difference between AIDS and HIV was often unclear and there were doubts about transmission routes and the degree of infectiousness of individuals under antiretroviral treatment. One participant questioned the existence of HIV, a belief that had been fostered through the use of Internet as a tool to fill information gaps.

*There is a video on the Internet that says that those drugs are bombs that kill people slowly*, *that they are an invention made by the pharmaceutical companies*, *by the US*. *I believed all that and was about to stop treatment*

[User 23, MSM]

Several participants mentioned that testing negative on repeated occasions generated feelings of invulnerability and contributed to perpetuating risky behaviours.

*Usually*, *when the test is negative*, *the majority of people I know*, *they don’t really change their behaviours after testing for HIV*. *Precisely because of that*, *because we think “so*, *I had the test and nothing*, *it is negative”*. *Then we think that we are invulnerable*

[User 20, MSM]

*I had negative results*. *I was happy*. *I walked out the door and did not ask anything because nothing ever happens to me*. *I told myself*: *They can say whatever they want but nothing happens*. *It has been many many years that I’m having sex with other men*, *and I’ve always played*, *and I am still negative*

[User 7, MSM]

### Connection to care and treatment: “We need time and staff, precisely what we haven’t got”

For most HPs, communicating an HIV+ test result was an emotionally charged experience for which they felt “always unprepared” irrespective of their experience and training courses received. Feelings of having done it “wrong” were aggravated in busy clinics because “you can’t explain that in two words”. In some facilities, an HIV+ result was an unexpected rare event and HPs were particularly unprepared to communicate a positive diagnosis for a severe disease, generally unrelated to the reasons that had originally prompted the medical consultation.

*My doctor saw the results and his face changed dramatically*. *He turned pale and his voice tone changed*. *My world fell apart and I burst into tears right there*, *in front of him*. *A nervous person cannot calm another nervous person*

[User 11, MSM]

Ensuring successful linkage to treatment “took its time” and the decree-law that restricted healthcare entitlements to undocumented migrants eight months before the initiation of this study had created confusion over actual entitlements to HIV treatment.

*Persons in irregular status don’t know who will cover the costs and health workers may not know because their orders have changed and they do not know what to do*. *Undocumented people are entitled to receive free HIV care and treatment because the decree law has exceptions*. *They should not be invoiced for the care received but in the health facilities some people know the instructions and others don’t*, *so some migrants receive an invoice and then they freak out*. *They fear that having a debt with the state will affect their possibilities to ever obtain legal residence in this country*

[HP 21]

The pathways to accessing treatment became increasingly intricate as illustrated by a young HIV+ undocumented resident who gratefully reported that the good will and coordinated effort of HPs at different institutions had granted him privileged access to treatment.

*They cared a lot about me*. *When I arrived there one said*: *he is the guy*. *I felt like I was the chosen one*. *It was very nice because I have no documents*, *I have no money*, *and just after getting there they were all taking care of me*

[User 4, MSM]

Many HPs stressed the importance of allocating sufficient time and resources to ensure adequate care and completion of the linkage to treatment process.

*At the end it is always the same*. *It’s all about resources*. *We are always on the front line*, *we remain active*. *The ideas flow*. *The problem is that there is no time to implement them*. *We could improve a lot*. *We have improved over the years because we are keen to it but at the end*, *what happens*? *You need time*: *time for the visit*, *time to do more*. *And that is what we are lacking*. *We are in very difficult times and with no resources*. *We need time and staff*, *precisely what we haven’t got these days*

[HP 5]

### Correct test results: “Professional”

For many users, testing at a health facility offered the greatest reliability standards and the attention received was positively valued as “professional”. The diagnostic tools employed offered maximum warranty of accuracy and procedures such as blood extraction, and provision of written records of test results were appreciated.

*They give you a copy of every result*. *Very elegant*. *At home I have a file named “analyses”*, *impressive*! *and then*, *if I have any doubt*, *I go to see my general practitioner and she gives me clear explanations*

[User 5, TW]

*I like to have my blood drawn*. *I like them to analyse it thoroughly*. *That way I feel reassured that it is really a “NO” because just with a prick*, *even if it is safe*, *I think*: *what if they got it wrong*? *I feel like I want to repeat it*. *But when I see the big syringe and they draw a lot of blood I feel well-served*

[User 1, TW]

### Confidentiality: “There are many holes”

Safeguarding confidentiality relating to HIV test results was of paramount importance for most of the HPs interviewed, but many were also sceptical and acknowledged that breaches of confidentiality occurred. There could be *holes* in information systems, users often attended primary care consultations with family members, some HPs “talked too much” and when cultural mediators originated from small communities, strict confidentiality could not be guaranteed.

The mediator may not know anyone (from the user’s community) and the issue of confidentiality may be credible or maybe he sees him every evening at the community association

[HP 3]

*I have all his information*. *I know everything about him*. *I know where he lives*, *phone numbers*, *the complete medical history*. *It is confidential (ironical tone) but I know where you live*

[HP 7]

*The access to users’ data is not completely closed*. *That can be helpful for certain issues*, *but there are many holes*

[HP 12]

For many users privacy could be better safeguarded at health facilities than at MSM-specific community-based testing services where it would be relatively easy to come across an acquaintance. Still, there were concerns that all databases containing personal information were linked and several feared being recorded in health facility files and subsequently deported because of their HIV+ and/or undocumented status. Sex workers were concerned about losing clients and one participant worried that his social security contributions would rise if he were found to be HIV+.

*If this information reaches the social security officers it will ruin my life completely*. *They know everything about you*. *It is on their computers*. *Your credit card number*, *your telephone number*. *All the data is linked*. *They know everything and I am afraid that they will raise my social security contribution and I will have to pay more taxes if I am HIV+*

[User 9, MSM]

*Why should I undergo an HIV test*? *They will expel me from this country if the test is positive*. *They will expel me from my job*. *I am enrolled in the social security system*, *what will happen to me if the test is positive*? *The first thing that would cross your mind would be to be expelled from Spain because there are many countries which restrict entrance to HIV+ people*

[User 21, MSM]

### Sexual minority stigma: “Fags are bad people”

Most study participants held ambivalent views and the widespread claim that HIV should be treated as a “normal” disease co-existed with explicit acknowledgement that it was exceptionally charged with stigma. A HP who said that HIV testing should be performed without asking for consent as a means to normalise the disease, later on explained that his HIV+ colleagues were treated in a different town for fear of stigma. Users originating from countries where “the government publicised that homosexuality was damaging for society” exhibited particularly high levels of self-stigma and often blamed sexual minorities for promiscuous and irresponsible sexual behaviour exacerbated by the use of sex-enhancement drugs. Many reported that HIV continued to be seen as a disease that affected “strange”, “careless” and “dirty-minded” people as a result of their free choice for deviant behaviours (“you get it if you want”). Several MSM participants referred to “fags” as “cochinos” (pigs), “disgusting”, and “irresponsible” people who spread the disease and prioritise their own sexual pleasure over others’ people’s rights to remain uninfected. Some foreign-born MSM severely criticised in moralising terms what they perceived as liberal sexual attitudes and openly effeminate manners of local MSM.

*The big difference between cancer and AIDS is that people get AIDS because they don’t control themselves*. *But a cancer is different; it is something you cannot control in your life*. *Do those people want to take care of themselves or not*? *If they don’t want to*, *then we will need to be class-conscious*, *look for all those who want to take care of themselves and send them to a part of the world and*, *on the other hand*, *send to a different part of the planet all those people who want to live with their diseases and their things*, *and free… you know*? *And… with no medicines*

[User 5, TW]

*They spend their time in saunas*, *infecting more people simply because they know they are also positive*. *Yes*. *Fags are bad… they are VERY bad*. *Just because they have it*, *they want everyone else to have it too*. *I am gay too but I am telling you*: *fags are very bad*

[User 4, MSM]

Enacted stigmatisation was commonly reported. Generally ascribed to their sexual orientation it was particularly severe in the case of TW and HIV+ people, and could overlap with other sources of stigma.

*Migrant*, *ugly*, *fat and HIV+*! *If you are attended by a person who is not keen on migrants because s/he thinks that public money should be used only for the Spaniards and in addition you have a very serious disease that you brought from your country of origin*, *then discrimination is greater*

[User 37, MSM]

## Discussion

PITC was conceptually acceptable for both users and providers and positively envisaged as a testing modality that would normalise HIV testing and promote earlier HIV diagnosis. For the majority of users interviewed, health facilities were convenient testing venues and the offer of an HIV test by a HP was appreciated. Many users reported that PITC was the testing modality that offered the greatest advantages in terms of convenience, reliability and confidentiality suggesting that it is a promising approach to promote testing amongst foreign-born sexual minorities who are reluctant to attend community-based services. However, HPs’ reports suggest that endorsement of and adherence to the 5Cs principles underpinning the WHO rights-based response to HIV varied widely across health settings. Counselling was often skipped, confidentiality could not be strictly safeguarded, and a few HPs acknowledged testing users for HIV without asking for consent. Linkage to treatment was a time-consuming process, further complicated by uncertainties and confusion in the case of undocumented migrants. In spite of—and sometimes because of—questionable adherence to some of the 5Cs principles, PITC continued to be acceptable for most users, suggesting that instances of poor adherence to ethical standards in HIV testing services could remain uncontested. Widespread claims that HIV testing should be normalised coexisted with blaming and moralising attitudes amongst both users and providers, which continued to frame HIV as an exceptional disease. The operational challenges identified point to shortcomings in the range of services that should be provided together with HIV testing [3], and suggest a call for caution in the implementation of PITC in contexts were persisting and overlapping sources of HIV stigma coexist with partial endorsement of the core principles underpinning the rights-based response to HIV.

The majority of foreign-born sexual-minority participants consulted in our study appreciated a HPs’ offer to test for HIV as a way of avoiding the embarrassment of “asking for it” and being qualified as both “promiscuous” and an “abuser” of the entitlements granted to them as foreigners. Our findings suggest that PITC was often framed as “good care” and that the medical context inherent in PITC facilitated the uptake of HIV tests [52]. Other studies of patients’ perceptions also suggest that PITC is generally acceptable to migrants and ethnic minorities [48,53,54]. In the UK, black patients were more likely to accept a rapid HIV test offer as part of a new patient heath check-up than other users [53]. In Finland, 92% of migrants from various socio-demographic backgrounds accepted the offer [54], and in Spain a PITC intervention that included migrants amongst other targeted populations was successful in reaching new foreign-born testers [48].

While generally acceptable, we identified a number of challenges in the operational implementation of PITC and varying degrees of adherence to the 5Cs principles that merit attention.

Informed consent procedures varied widely across health settings. In certain sites these seemed to be systematically applied while in others a *culture* of testing users for HIV without informing them seemed to prevail. Similar concerns have been raised in other studies [55]. A remarkable finding of our study is that PITC continued to be acceptable for many of the users interviewed in spite of this shortcoming. In fact, several deemed compulsory testing to be acceptable on public health grounds. Along similar lines, although pre-counselling and post-counselling sessions for HIV-negative cases were frequently skipped, failure to provide counselling was not judged negatively by users. On the contrary, many appreciated facility-based testing precisely because it allowed them to bypass counselling. However, we also identified important gaps in knowledge and some participants’ attempts to cover these through alternative and sometimes misguided sources of information derived from the internet. As HIV testing is increasingly offered, it is important to ensure that all testers are adequately informed about the disease and its treatment. Counselling formats, contents and procedures should be tailored to meet the needs of frequent testers and those of users who attend health facilities for reasons unrelated to HIV.

Communicating an HIV+ result posed a major emotional burden for many HPs. Linking HIV+ patients to care and treatment, especially undocumented migrants, was a time-consuming process that HPs navigated amidst confusion about actual health care entitlements for undocumented migrants in Spain. The costs in terms of the time that HPs need to successfully link patients to treatment should be accounted for as part of PITC programmes. Special attention should be paid to facilities in areas where the largest number of new HIV+ cases are likely to be identified. Clear information about migrants’ entitlements to treatment should be provided so that perceived barriers for accessing treatment do not hinder HIV test uptake.

For the majority of users interviewed, health facilities were trustworthy institutions that offered the best guarantee of accuracy and privacy. However, HPs acknowledged that it was impossible to guarantee strict confidentiality and some users feared the negative legislative, occupational or fiscal consequences of an eventual HIV+ result that was not kept confidential (“all the data is linked”). Our findings are consistent with a literature review showing that fears of confidentiality gaps are amongst the most common reasons for not testing for HIV in high income countries [33]. As PITC is more broadly implemented, it is of paramount importance to ensure sustained trust in the health care system. Strategies deployed to communicate test results that “cheat users a bit” (E.g. asking an HIV+ user for consent to test for HIV *after* the test has already been performed) could undermine this crucial factor to effectively promote HIV uptake amongst migrants [56,57].

Universal PITC policies were originally conceptualised to routinise and hence destigmatise the testing process through non-targeted testing but adherence to universal PITC guidelines is generally patchy [30,58–63]. HIV test coverage remains low in many locations and the test offer is often targeted as opposed to routinely applied. Concerns that screening will still be targeted at certain groups based on HPs’ judgement of their behaviours, sexuality or ethnicity have been raised in previous qualitative studies [32]. In practice, a blurred line separates universal from targeted PITC approaches. While users in our study appreciated the offer of the test and were generally not concerned about “being targeted”, it is important to identify potential negative impacts on rates of acceptance of HIV test offers that may result from broader implementation of PITC approaches, and to monitor perceptions amongst other migrant populations whose views may differ. In some settings the offer of the test is generally accepted by migrant and ethnic minorities [54,62] but in others the suitability of the approach to reach ethnic minorities has been questioned. In a community health centre of the Bronx in New York, for example, 65% of the patients that had been offered a test—mostly black and Hispanic—declined the offer [51]. If the persons most likely to be infected are also those most likely to refuse the offer to test for HIV, the suitability of PITC to reduce late diagnosis will be compromised. The failure to identify new HIV infections in the Bronx study, which is amongst the areas with the highest HIV prevalence in New York, illustrates the risk of failure of PITC approaches if individuals most at risk decline the offer to test, no new cases are identified, and HPs develop “testing fatigue” within a vicious circle that would make them reluctant to sustain the extra effort of continuing to offer the HIV test in accordance with the 5Cs [51]. Even in settings where high acceptance rates amongst migrants are reported, it should be considered that language barriers and scarcity of interpreters could partly explain the low HIV test refusal rates in populations with poor understanding of the local language [54]. The limited power that certain persons may have to decline HIV test offers fed the debate on the implementation of PITC in sub-Saharan African settings [25,64] and should not be overlooked as a potential explanation in concentrated epidemics. Patient-provider power imbalances may be particularly relevant for sexual and ethnic minority users in contexts where health care entitlements are granted and retrieved by decree-law and their actual access to services ultimately depend on the good-will and perseverance of health professionals.

In our study widespread claims that HIV testing should be normalised coexisted with blaming and moralising attitudes that continued to frame HIV as an exceptional disease. This is in agreement with studies conducted in generalised epidemics [65,66]. The high levels of internalised sexual minority stigma that we identified have been associated with poor HIV test uptake in other settings [67,68] and could explain the frequently reported preference for ordinary health venues as opposed to mobile HIV testing units or testing sites “for gays”. Still, it should be considered that many MSM prefer to test in community-based settings [69] and mobile units are suitable testing sites for those less concerned about stigma [70], which calls for continued support for different HIV testing modalities.

As PITC is more broadly implemented, robust monitoring systems are needed to allow for an early identification of challenges and timely introduction of corrective measures. Key populations may be more susceptible to coerced testing so it is particularly important that HP emphasize the voluntary nature of HIV testing and the user’s right to decline [71]. As reiterated by WHO and UNAIDS, endorsement of PITC is not an endorsement of coercive HIV testing and all individuals should receive sufficient information to make an informed and voluntary decision to be tested [3,6,49]. Larger studies based on exit interviews with patients would help us to understand the extent to which consent is actually skipped and how procedures are applied in practice. The wide array of participatory process evaluation tools developed as a response to the HIV emergency in countries with generalised epidemics [72] could be adapted to inform HIV testing recommendations in Europe. The many-layered challenges faced by HPs should be considered for a better understanding of how they pragmatically manage the tensions between the theoretical constructs implicit in HIV testing guidelines and the day-to-day constrains they face, such as lack of time and insufficient preparation to deliver HIV+ test results. International guidance recommends that the counselling skills of HPs providing HIV testing services to foreign born vulnerable populations should be strengthened, together with their capacities to obtain informed consent, protect confidentiality and not discriminate [71]. While guidelines and training programmes targeting professionals’ knowledge and skills are needed, it is also essential to address structural limitations and broader institutional cultures [73]. The exceptionalism of HIV testing was the response—and not the cause—of the exceptional stigma ascribed to the disease, so it should not be expected that normalising testing procedures would in itself normalise HIV. Ethical scrutiny and human rights monitoring is needed to warrant adequate implementation of the approach.

Our results should be interpreted with caution. Most participants had undergone HIV testing at health facilities or mobile units so we only gathered superficial data about other testing modalities, including testing at recreational venues and pharmacies, and self-testing. Complacency bias might have resulted from the association of this research to a major public institution providing medical services, including ART treatment. However this was minimised by recruiting participants through community associations and conducting the interviews away from health facilities and in two different towns. We did not provide any remuneration to study participants, which may have fostered the participation of persons holding more positive attitudes. The views of most vulnerable persons are under-documented, as they are probably less likely take part in time-consuming and demanding research. Nonetheless, few qualitative studies have so far focused on MSM from ethnic minorities, and in spite of the extraordinary HIV burden carried by transgender women, this is amongst the few studies that disaggregates this population from the broader MSM population. We acknowledge that we did not reach data saturation, mostly because recruiting and engaging TW was more time-consuming than initially envisaged and it was not feasible to continue to gather data within the time-scale of this study. Clearly, further studies are needed to gather additional insights into the specific needs of a neglected population whose needs are persistently overlooked in spite of their enormous vulnerability to HIV acquisition, and the detrimental social consequences related to it [45,74].

## Conclusion

PITC offers a good opportunity to reach sexual minority migrants holding high levels of internalised stigma during routine medical consultations but extra efforts are needed to safeguard the rights of the most vulnerable in contexts where overlapping stigmas coexist with limited endorsement of right-based principles. PITC policies should be coupled with adequate funding and robust process evaluation studies.

## Ethical Approval

All procedures were in accordance with the ethical standards of the institutional and/or national research committee and with the 1964 Helsinki declaration and its later amendments or comparable ethical standards. Informed consent was obtained from all individual participants included in the study. Ethical approval was obtained from the Hospital Clinic-University of Barcelona Ethical Committee Board.
